# Adenovirus infection promotes the formation of glioma stem cells from glioblastoma cells through the TLR9/NEAT1/STAT3 pathway

**DOI:** 10.1186/s12964-020-00598-7

**Published:** 2020-08-26

**Authors:** Jian Zang, Min-Hua Zheng, Xiu-Li Cao, Yi-Zhe Zhang, Yu-Fei Zhang, Xiang-Yu Gao, Yuan Cao, Mei Shi, Hua Han, Liang Liang

**Affiliations:** 1 State Key Laboratory of Cancer Biology, Fourth Military Medical University, Xi’an, 710032 China; 2 Department of Biochemistry and Molecular Biology, Fourth Military Medical University, Chang-Le Xi Street #169, Xi’an, 710032 China; 3 Department of Radiation Oncology, Xijing Hospital, Fourth Military Medical University, Chang-Le Xi Street #169, Xi’an, 710032 China; 4 Department of Medical Genetics and Developmental Biology, Fourth Military Medical University, Xi’an, 710032 China

**Keywords:** Glioma stem cells, Adenovirus, DAMP, NEAT1, TLR9

## Abstract

**Background:**

Glioma stem cells (GSCs) are glioma cells with stemness and are responsible for a variety of malignant behaviors of glioma. Evidence has shown that signals from tumor microenvironment (TME) enhance stemness of glioma cells. However, identification of the signaling molecules and underlying mechanisms has not been completely elucidated.

**Methods:**

Human samples and glioma cell lines were cultured in vitro to determine the effects of adenovirus (ADV) infection by sphere formation, RT-qPCR, western blotting, FACS and immunofluorescence. For in vivo analysis, mouse intracranial tumor model was applied. Bioinformatics analysis, gene knockdown by siRNA, RT-qPCR and western blotting were applied for further mechanistic studies.

**Results:**

Infection of patient-derived glioma cells with ADV increases the formation of tumor spheres. ADV infection upregulated stem cell markers and in turn promoted the capacities of self-renewal and multi-lineage differentiation of the infected tumor spheres. These ADV infected tumor spheres had stronger potential to form xenograft tumors in immune-compromised mice. GSCs formation could be promoted by ADV infection via TLR9, because TLR9 was upregulated after ADV infection, and knockdown of TLR9 reduced ADV-induced GSCs. Consistently, MYD88, as well as total STAT3 and phosphorylated (p-)STAT3, were also upregulated in ADV-induced GSCs. Knockdown of MYD88 or pharmaceutical inhibition of STAT3 attenuated stemness of ADV-induced GSCs. Moreover, we found that ADV infection upregulated lncRNA NEAT1. Knockdown of NEAT1 impaired stemness of ADV-induced GSCs. Lastly, HMGB1, a damage associated molecular pattern (DAMP) that triggers TLR signaling, also upregulated stemness markers in glioma cells.

**Conclusion:**

ADV, which has been developed as vectors for gene therapy and oncolytic virus, promotes the formation of GSCs via TLR9/NEAT1/STAT3 signaling.

Video abstract

## Background

Cancer stem-like cells (CSCs) are considered to be responsible for cancer recurrence and metastasis because of their ability to resist conventional chemo- and radio-therapies of cancer and re-form new tumors [1–3]. CSCs have been identified in many cancers, but their origins have been elusive [4–9]. While epigenetic modifications, developmental pathways, metabolic reprogramming and so on have been implicated in the formation and maintenance of CSCs [10–15], recent studies have provided evidence that stimulations from tumor microenvironment (TME) can evoke the stemness of “ordinary” cancer cells [16–18], as manifested by spherical growth and multi-lineage differentiation potentials, expression of stemness markers and transcription factors, and stronger tumorigenic capacity in immune-compromised hosts [18, 19]. For example, hypoxia, which is a fundamental characteristic of TME in solid tumors, promotes the formation and inhibits the differentiation of CSCs in many tumor models [20, 21]. Inflammation, which is another hallmark of cancer, generates a large array of cytokines that promote stemness of cancer cells [22, 23]. Moreover, some of the damage-associated molecular patterns (DAMPs) such as the high mobility group box 1 (HMGB1) derived from tumor cells or TME could also promote the CSCs phenotype via innate immune signaling [24–26]. Mechanistically, toll-like receptor 9 (TLR9)/myeloid differentiation primary response gene 88 (MYD88) pathway [27–29], signal transducer and activator of transcription 3 (STAT3) [30], as well as some long non-coding RNA such as the nuclear enriched abundant transcript 1 (NEAT1) [31–34], have been implicated. However, the identity and mechanisms of TME-derived cues in promoting CSCs formation have not been completely understood.

Glioblastoma multiforme (GBM) is the most common malignant brain tumor in adults with very poor prognosis [35]. Multiple studies have confirmed the existence of CSCs in glioma, or glioma stem cells (GSCs), which are capable of self-renewal, extensive proliferation, and multi-lineage differentiation [9, 14, 19]. These studies have demonstrated that the rare population of GSCs is necessary and sufficient to initiate, maintain, and recapitulate the phenotype of original glioma in immune-compromised mice, and when GSCs are eliminated from the bulk tumor mass, tumor growth is inhibited [36, 37]. Therefore, GSCs could play a pivotal role in glioma development in human, and these cells seem to be a promising target for glioma therapies [38]. However, although there is strong evidence that GSCs contribute to tumor propagation and treatment resistance, the potential mechanisms underlying the acquisition of stemness by glioma cells to transit into GSCs remain to be elucidated.

Adenovirus (ADV) has been progressively modified to satisfy the needs of human gene therapy and oncolytic virotherapy of cancer [39, 40]. Because ADV DNA usually does not integrate into host genome, ADV is generally considered safe in gene therapies, although some viral components invoke innate immune responses [41]. To our knowledge, no reports have shown that ADV could promote CSCs. In this study, we found that infection of adenovirus itself promoted the formation of tumor spheres from glioma cells, a sign of acquisition of stemness. We further revealed that ADV infection could indeed promote the formation of GSCs from patient-derived glioma cells. Considering that ADV and other virus-based vectors are increasingly employed in developing gene therapy and oncolytic viral therapy, our data emphasize a potentially undesirable effect of these vectors, namely, increasing the risk of formation of CSCs.

## Materials and methods

### Human samples and cell culture

GBM tissue samples were obtained from patients accepting surgical resection of tumors at the Department Neurosurgery, Xijing Hospital, Fourth Military Medical University. The diagnosis was confirmed by pathology. Informed consent was obtained from each subject involved in this study. The use of human tissues was approved by the Ethics Committee, Xijing Hospital.

To culture primary GBM cells, fresh tumor tissues were dissociated into single cell suspensions by mechanical grinding. Cells derived from two patients (named as FMXJ-1 and FMXJ-2, respectively) were cultured in Dulbecco’s modified Eagle’s medium (DMEM)/F12 (1:1) (Invitrogen, Carlsbad, CA) supplemented with 10% fetal bovine serum (FBS) and 1% penicillin–streptomycin solution. Cells were passaged routinely with the same medium as stocks of primary GBM cells. To culture GSCs, the primary GBM cells were cultured under the neurosphere condition in DMEM/F12 containing 20 ng/ml epidermal growth factor (EGF, Peprotech, Rocky Hill, NJ), 20 ng/ml basic fibroblast growth factor (bFGF, Peprotech), B27 (1:50, Invitrogen) and N2 (1:100, Invitrogen) for 7 days to obtain tumor spheres (with > 50 cells) [39]. For re-plating, spheres were mechanically dispersed, counted, and cultured as above for 7 days. For differentiation, spheres were dissociated into single cells and cultured in DMEM/F12 median containing 10% FBS for 5 days. In some cases, cells were infected by ADV for 8 h before plating. In some experiments, primary GBM cells were cultured in the presence of CpG oligodeoxynucleotides (2 mM, InvivoGen, San Diego, CA) for 24 h, HMGB1 (1 μg/mL, GenScript, Piscataway, NJ) for 48 h, or Stattic, a STAT3 inhibitor (Calbiochem, San Diego, CA) for 4 h [40], respectively. Cells were then transferred to GSC medium and cultured for 7 days, and tumor spheres were counted.

A172 and T98G glioma cell lines were purchased from ATCC (Manassas, VA), and cultured in DMEM supplemented with 10% FBS and 1% penicillin–streptomycin solution. All cells were cultured in a standard culture incubator with 5% CO_2_ in air and 100% relative humidity at 37 °C.

### Viral infection and transfection of cells

ADV particles, which were generated by co-transfecting HEK293A cells with pAdTrack-CMV and pHBAd-BHG using the Adeasy adenovirus system, were purchased from HANBIO Biotechnology (Shanghai, China) with viral titers of 1.26 ╳ 10 [10] PFU/ml. For infection, primary GBM cells were incubated with viral particles at 400 multiplicity of infection (MOI), and the medium was changed 8 h later. siRNA against different genes and negative control siRNA (NC) were purchased from RiboBio (Guangzhou, China). Primary GBM cells were transfected with 10 nM of siRNA using Lipofectamine 2000 (Life Technologies) following the manufacturer’s protocol. Forty-eight hours after the transfection, cells were re-plated for tumor sphere assay, or total RNA or protein was extracted from the transfected cells for further experiments.

### Tumor growth in vivo

BABL/c-A nude mice at 4–6 weeks of age were used for intracranial xenograft tumor inoculation according to a published protocol [41]. Briefly, tumor cells were labeled with a luciferase fusion reporter by lentivirus-mediated transfection. Different number of cells (500, 5000, 10,000) in 3 μl of DMEM/F12 medium were injected into the brain hemisphere of mice under the navigation of a murine brain stereotaxic apparatus (RWD68000, RWD Life Sciences Co., Ltd., Shenzhen, China). Tumor growth was monitored by intracranial bioluminescence using an IVIS Kinetic Imager (PerkinElmer, Waltham, MA). Tumors were dissected from the mouse brain and fixed in 10% formalin. Samples were embedded in paraffin, and sections were made for immunohistochemistry or hematoxylin and eosin (H&E) staining. All animal experiments were approved by the Animal Experiment Administration Committee of the Fourth Military Medical University.

### Immunofluorescence

Cells cultured on coverslips were fixed in 4% paraformaldehyde (PFA) for 10 min and rinsed with PBS for three times, followed by permeabilization with 0.2% Triton X-100 for 10 min. Samples were blocked with 1% bovine serum albumin (BSA) for 30 min and incubated with primary antibodies overnight at 4 °C. Cells were then incubated with Cy2-conjugated secondary antibodies for 1 h. Washing with PBS was performed between each staining step. Nuclei were counter-stained with Hoechst for 5 min. The antibodies used included mouse anti-mitogen associated protein 2 (MAP2, 1:1000, Sigma, St. Louis, MO), rabbit anti-gial fibrilling acidic protein (GFAP, 1:500, Sigma), mouse anti-O4 (1:100, Sigma), Cy2-conjugated donkey anti-mouse (1:500) and Cy2-conjugated donkey anti-rabbit (1:500, Jackson ImmunoResearch, West Grove, PA). Samples were examined under a fluorescence microscope (FV-100, Olympus, Japan).

### Flow cytometry

Single cell suspensions were prepared and incubated with a rabbit anti-CD133 (1:50, Proteintech) for 30 min at 4 °C in dark. Cells were washed and then stained with FITC-conjugated goat anti-rabbit secondary antibody, followed by FACS analysis using a FACS Calibur™ flow cytometer (BD Immunocytometry Systems, Franklin Lakes, NJ). Dead cells were excluded by propidium iodide (PI) staining. The acquired data were analyzed with FlowJo vX.0.6 software (Tree Star Inc., Ashland, OR).

### Quantitative Reverse Transcription-Polymerase Chain Reaction (RT-PCR)

Total RNA was extracted using the Trizol reagent (Invitrogen) and was reverse-transcribed into cDNA with a kit (Takara, Dalian, China). Quantitative (q)PCR was performed on an ABI PRISM 7500 Real-time PCR system (Life Technologies, Waltham, MA) using a SYBR Premix Ex Taq Kit (Takara), with β-actin as a reference control. Primers are listed in supplementary Table S1. All RT-qPCR experiments were performed in triplicates for at least 3 times.

### Western blotting

Cells were lysed and soluble proteins were extracted using the radio immunoprecipitation assay (RIPA) buffer (Beyotime, Shanghai, China) containing a protease inhibitor cocktail and sodium orthovanadate (Santa Cruz Biotechnology, Dallas, TX). Protein concentration was determined using a BCA protein assay kit (Pierce Biotechnology, Rockford, IL). Protein samples were then run on sodium dodecyl sulfate-polyacrylamide gel electrophoresis (SDS-PAGE) with 10% polyacrylamide, and electro-transferred onto polyvinyl difluoride (PVDF) membranes (Millipore, Billerica, MA). The membranes were probed with specific primary and secondary antibodies, and developed with chemiluminescence (ECL, Thermo Fisher) using the ChemiDoc Touch Imaging System (BioRad). Quantification of bands was achieved by a densitometry, with β-actin as a reference control. Specific primary antibodies against the following proteins were used: c-MYC (SAB, 1:1000), SOX2 (R&D Systems, 1:1000), OCT4 (Cell Signaling, 1:1000), NANOG (SAB, 1:1000), STAT3 (SAB, 1:1000), pSTAT3 (SAB, 1:1000), MYD88 (R&D Systems, 1:1000), β-actin (Santa Cruz Biotechnology, 1:2000). Species-specific horseradish peroxidase (HRP)-conjugated anti-rabbit IgG (Jackson ImmunoResearch) and HRP-conjugated anti-mouse IgG (Jackson ImmunoResearch) were used as secondary antibodies.

### Bioinformatics

mRNA expression datasets and the associated clinical information were downloaded from TCGA (http://xena.ucsc.edu/getting-started/) and CGGA (http://www.cgga.org.cn). Gene set enrichment analysis (GSEA) was performed with the GSEA v2.0 software (Broad Institute of MIT, Massachusetts Institute of Technology). Probed gene sets were taken without further from the indicated publications, and downloaded from the KEGG pathway database (gseaftp.broadinstitute.org://pub/gsea/gene_sets_final/c2.cp.kegg.v6.2.symbols.gmt). The normalized enrichment scores (NES) with *P* values < 0.05 and false discovery rates (FDR) < 0.25 were considered statistically significant.

### Statistical analysis

Statistical analysis was performed with the GraphPad Prism 6 software. All the results were presented as the mean ± standard error of mean (SEM). Comparisons between groups were performed using unpaired, two-tailed, Student’s t-test and Analysis of Variance (ANOVA) with 95% confidence interval. Survival analysis was calculated using Kaplan-Meier curves (log rank test). *P* < 0.05 was considered statistically significant.

## Results

### ADV infection promotes the formation of GSCs in culture

**Fig. 1:**
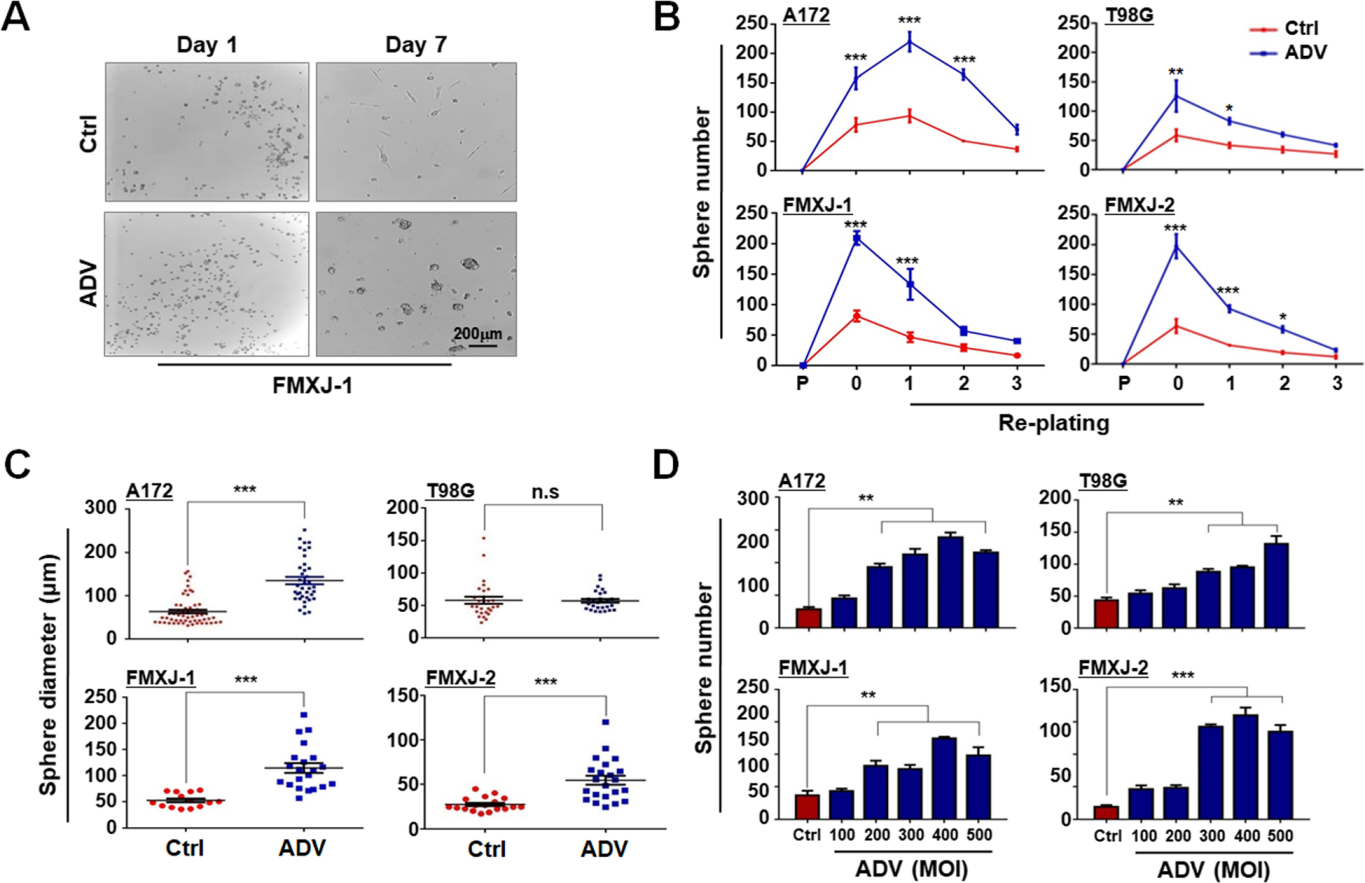
ADV infection promotes tumor sphere formation by glioma cells. **a** Primary GBM cells (FMXJ-1) were infected with ADV for 8 h, and then cultured under the neurosphere condition for 7 days and photographed. **b** Primary and lined glioma cells (P) cultured under ordinary condition without the sphere supplements). Cells were infected and cultured as in (A) for 7 days (re-plating 0). Spheres were then re-plated serially every 7 days for 3 times (as re-plating generation 1, 2, and 3, respectively). Number of tumor spheres was counted on each generation. Cell not infected with ADV were used as controls. **c** Diameter of spheres on day 7 was measured. **d** Primary and lined glioma cells were infected with different amounts of ADV (MOI) and cultured under the neurosphere condition for 7 days. Number of tumor spheres was counted. Data are represented as mean ± SEM, *n* = 6. *, *P* < 0.05; **, *P* < 0.01; ***, *P* < 0.001; n.s, not significant

In an attempt to ectopically express exogenous genes in human primary glioma cells using ADV-mediated transfection, we happened to find that infection of ADV itself promoted the formation of tumor spheres in culture (Fig. 1a, supplementary Figure S1). To confirm the phenomenon and test the re-plating capacity of the spheres, we infected another stock of patient-derived primary glioma cells and two glioma cell lines with ADV, and re-plated spheres every 7 days for 3 passages. The result showed that sphere formation was increased significantly from the ADV-infected cells, and this increased capacity of sphere formation was maintained for two more passages (Fig. 1b). The diameter of spheres increased significantly in the ADV-infected groups except for T98G (Fig. 1c). We also quantitatively tested the sphere formation by primary and lined glioma cells infected with ADV at different MOI. The results showed that the number of spheres increased proportionally with the increase of MOI (Fig. 1d). These data suggested that infection of ADV could promote stemness of glioma cells.

### ADV infection induces the transformation from non-GSCs to GSCs

**Fig. 2:**
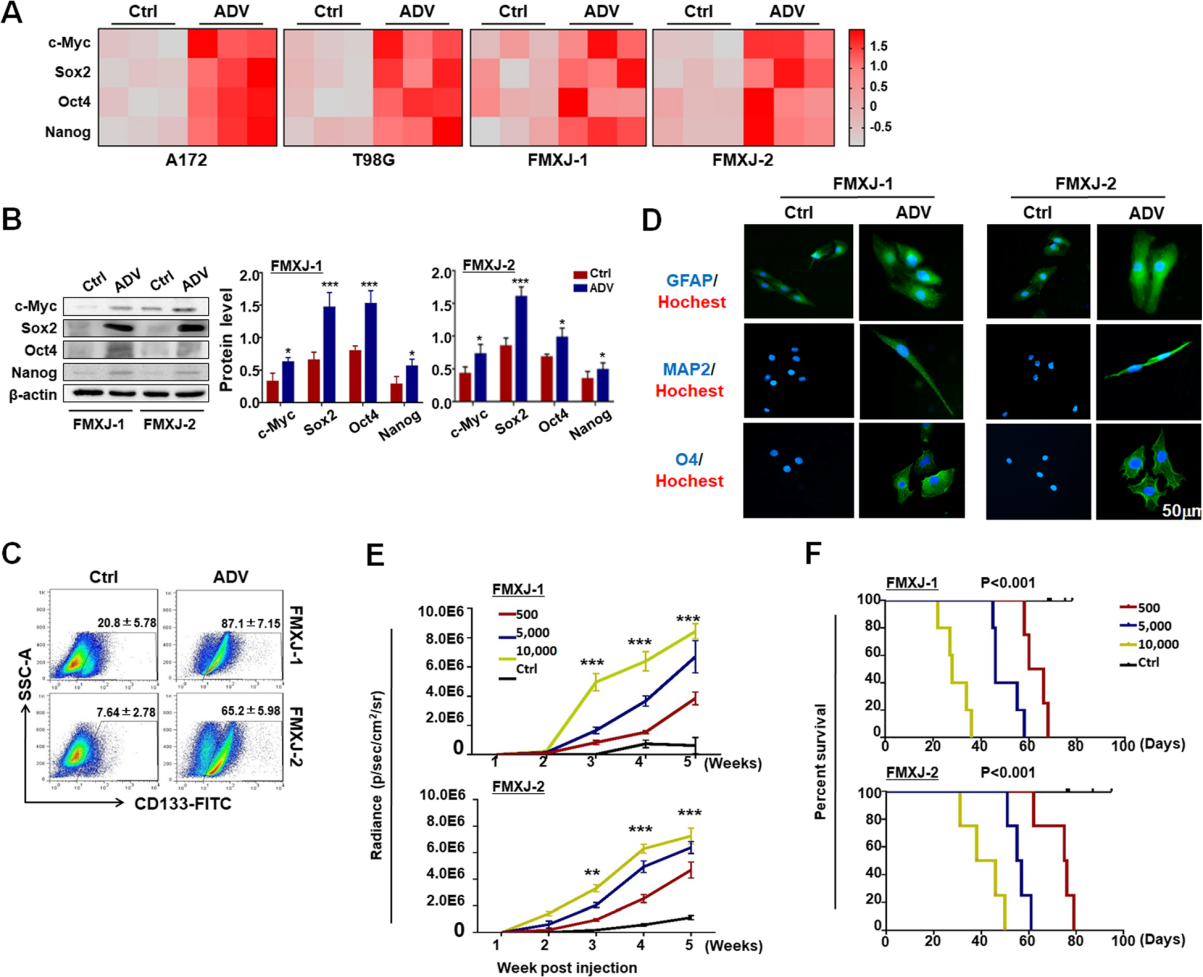
Characterization of tumor spheres derived from ADV-infected primary glioma cells. **a** Expression of stemness-related transcription factors in primary or lined glioma cells after ADV infection was determined by RT-qPCR, and represented as a heatmap (*n* = 3). **b** Expression of stemness-related transcription factors in primary glioma cells after ADV infection was determined by western blotting. **c** Tumor spheres from ADV-infected or uninfected primary glioma cells (Ctrl) were analyzed by FACS for CD133 (*n* = 6). **d** Tumor spheres derived from ADV-infected or control primary glioma cells were subjected to differentiation culture for 7 days. The expression of markers of astrocytes (GFAP), neurons (MAP-2), and oligodendrocytes (O4) was determined by immunofluorescence, and counter-stained with Hoechst (n = 3). **e, f** In vivo tumorigenic capacity of ADV-induced GSCs. ADV-induced tumor spheres and primary glioma cells were labeled with luciferase by lentivirus. Different numbers of GSCs (500, 5000, 10,000) and primary glioma cells (10,000, as Ctrl) were intracranially inoculated in nude mice, and bioluminescence imaging was employed to follow tumor growth at different time points **e**. The survival of mice was plotted simultaneously **f**. Data are represented as mean ± SEM, *n* = 5. **, *P* < 0.01.***, *P* < 0.001

To confirm the stemness of tumor spheres derived from glioma cells after ADV infection, we performed the following experiments. First, primary and lined glioma cells were infected with or without ADV, and the expression of pluripotency factors c-MYC, SOX2, OCT4 and NANOG were determined by RT-qPCR and western blotting. The result showed that ADV infection strongly upregulated these pluripotency factors at both mRNA and protein levels (Fig. 2a, b). The mRNA level of EpCAM also elevated after adenovirus infection (data not shown). Second, we determined the expression of the stemness marker CD133 using flow cytometry, and the result showed that ADV infection significantly increased the population of CD133^+^ cells in the ADV-infected glioma cells (Fig. 2c). Third, we tested the multi-differentiation potential of tumor spheres from ADV-infected glioma cells by adherent culture in the presence of serum. Immunofluorescence showed that these tumor spheres were able to differentiate into GFAP^+^ astrocytes, MAP2^+^ neurons, and O4^+^ oligodendrocytes (Fig. 2d). Lastly, we performed xenotransplantation assay to investigate the in vivo tumorigenic ability of tumor spheres derived from ADV-infected glioma cells (supplementary Figure S2A). Single-cell suspensions were prepared from tumor spheres of ADV-infected glioma cells. Different numbers (500, 5000, 10,000) of luciferase-labeled cells were injected intracranially into the brain hemisphere of nude mice under stereotaxic navigation, with 10,000 of primary glioma cells as a control. Bioluminescence imaging revealed that cells from tumor spheres initiated tumor growth with significantly higher efficiency than that of primary glioma cells (Fig. 2e, supplementary Figure S2B, S2C). The survival of mice was negatively correlated with the number of inoculated cells derived from tumor spheres (Fig. 2f). In a nutshell, these data indicated that tumor spheres from ADV-infected glioma cells possess the characteristics of GSCs.

### TLR9 is required for ADV-induced GSC formation

**Fig. 3:**
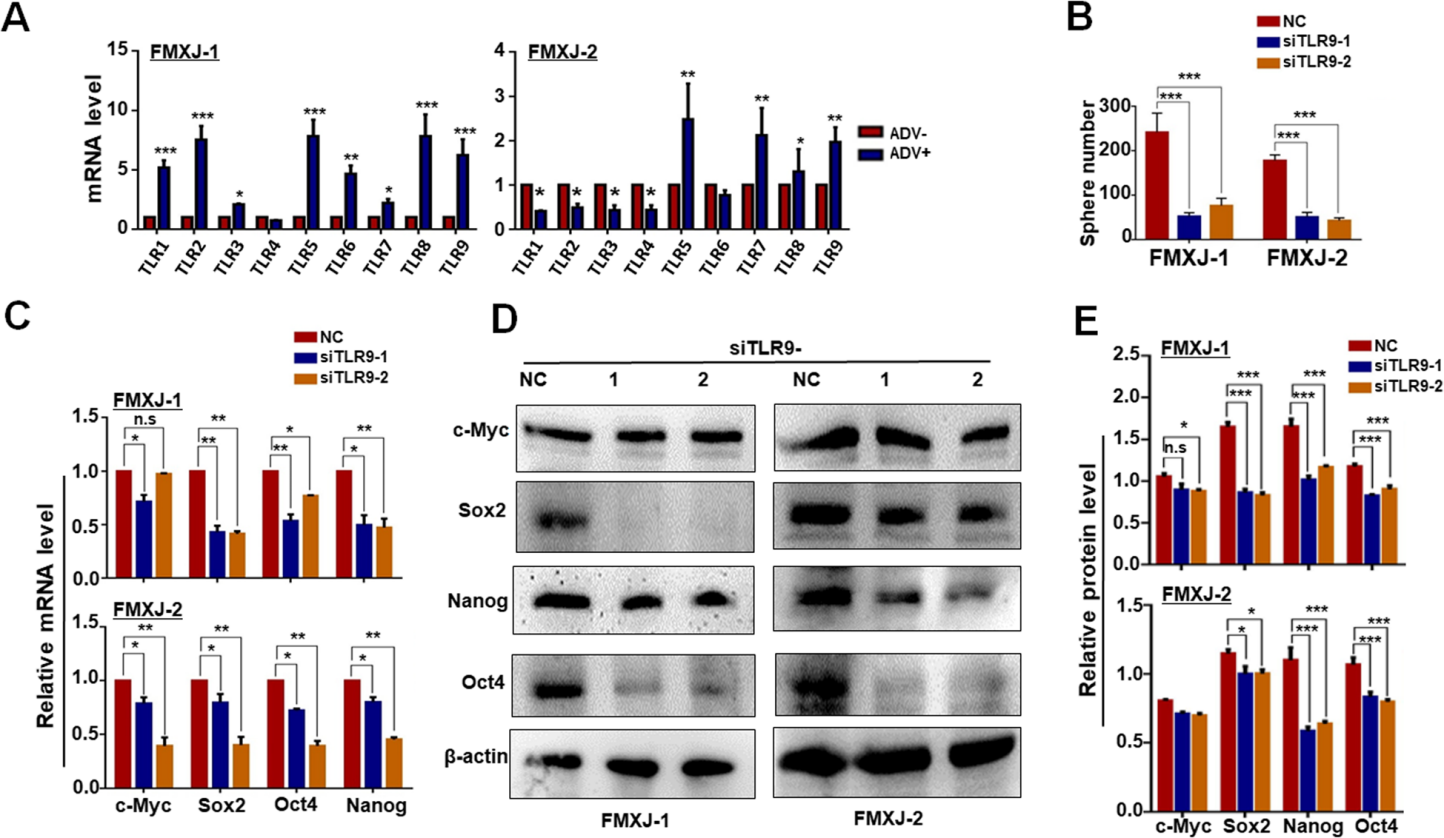
Knockdown of TLR9 compromises ADV-induced GSCs formation from primary glioma cells. **a** The expression of TLRs in tumor spheres from ADV-infected (ADV+) and un-infected (ADV-) primary glioma cells was determined by RT-qPCR. **b** Primary glioma cells were infected with ADV and transfected with siRNAs to TLR9, and then cultured under the neurosphere condition. The formation of tumorsphere was determined. **c-e** Primary glioma cells were infected with ADV and transfected with siRNAs to TLR9 (NC, negative control. 1, siRNA-1. 2, siRNA-2). The expression of stemness-related transcription factors including c-MYC, SOX2, NANOG, and OCT4 was determined by RT-qPCR **c** and western blotting **d, e**, with β-actin as a reference control. Data are represented as mean ± SEM, *n* = 6. *, *P* < 0.05. **, *P* < 0.01. ***, *P* < 0.001. n.s, not significant

We then set out to identify potential signaling pathways mediating ADV-induced GSC formation. Quantitative RT-PCR showed that cellular DNA sensors STING, AIM2 and cGAS were not consistently up-regulated in the two stocks of human primary glioma cells upon ADV infection (supplementary Figure S3A) [42, 43]. In addition, some TLRs could also play a role in recognition of viral DNA and/or other components [42, 44]. RT-qPCR showed that TLR5, 7, 8, and 9 were consistently upregulated in the two stocks of human primary glioma cells after ADV infection (Fig. 3a). Because previous studies have demonstrated that TLR9 is a DNA sensor to recognize the ADV DNA and is involved in GSCs and other CSCs [27–29], we then focused our further study on TLR9. We synthesized siRNAs targeting TLR9 (supplementary Figure S3B, S3C), and transfected ADV-infected primary glioma cells with these siRNAs. Tumor sphere formation was attenuated significantly by the TLR9 siRNAs compared with the negative control (Fig. 3b; supplementary Figure S3D). Consistently, the expression of stemness-related transcription factors including c-MYC, SOX2, NANOG, and OCT4 was reduced as determined by RT-qPCR and western blotting (Fig. 3c-e). These results suggested that ADV infection of primary glioma cells was likely to promote GSCs formation through the TLR9 pathway.

### ADV infection promotes GSC formation via the MYD88-STAT3 pathway downstream to TLR9

**Fig. 4:**
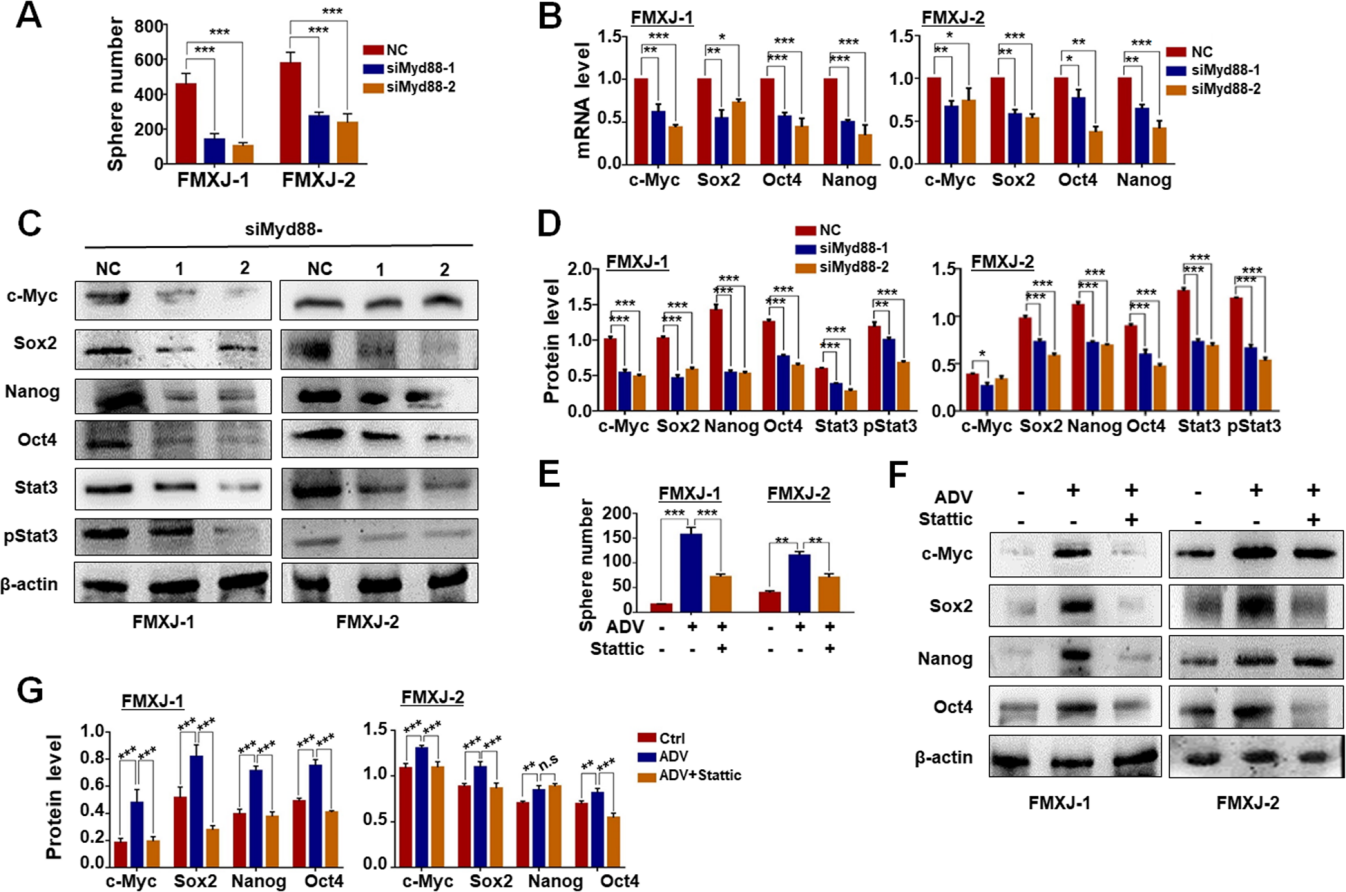
STAT3 downstream to MYD88 is required for ADV-induced GSCs formation from primary glioma cells. **a** Primary glioma cells were infected with ADV and transfected with siRNAs to MYD88, and then cultured under the neurosphere condition. The formation of tumorsphere was determined. **b-d** Primary glioma cells were infected with ADV and transfected with siRNAs to MYD88 (NC, negative control. 1, siRNA-1. 2, siRNA-2). The level of stemness-related transcription factors, STAT3 and phosphorylated STAT3 was determined by RT-qPCR **b** and western blotting **c, d**, with β-actin as a reference control. **e** Primary glioma cells were infected with ADV, and cultured under the neurosphere condition in the presence of the STAT3 inhibitor STATTIC (2μM). The formation of tumorsphere was determined. **f, g** Primary glioma cells were infected with ADV, and cultured in the presence of the STAT3 inhibitor STATTIC. The expression of stemness-related transcription factors including c-MYC, SOX2, NANOG, and OCT4 was determined by western blotting, with β-actin as a reference control. Data are represented as mean ± SEM, *n* = 6. *, *P* < 0.05. **, *P* < 0.01. ***, *P* < 0.001. n.s, not significant

TLR9 likely promotes stemness in cancer cells via MYD88 and STAT3 [22, 30, 44]. Western blotting showed that the level of MYD88 decreased in primary glioma cells after siRNA targeting TLR9 (supplementary Figure S3E). Data from TCGA and CGGA database showed that higher levels of MYD88 and STAT3 correlated with a decrease in median survival of GBM patients (supplementary Figure S4A, S4B). Western blotting showed that the level of MYD88, STAT3 and phosphorylated STAT3 increased in primary glioma cells after ADV infection (supplementary Figure S4C, S4D). To evaluate whether ADV induced stem-like transformation via MYD88, we synthesized siRNA targeting MYD88 (supplementary Figure S3B, S3C), and transfected ADV-infected primary glioma cells. The result showed that transfection of MYD88 siRNA significantly reduced tumor sphere formation from GBM cells infected with ADV (Fig. 4a). Meanwhile, RT-qPCR and western blotting showed that MYD88 siRNA downregulated the expression of c-MYC, SOX2, OCT4, and NANOG at both mRNA and protein levels (Fig. 4b-d). The level of total STAT3 and phosphorylated STAT3 decreased also when ADV-infected glioma cells were transfected with MYD88 siRNA (Fig. 4c, d, supplementary Figure S3F). To evaluate whether STAT3 activation was required for ADV-mediated tumor GSC formation, primary glioma cells were infected with ADV and cultured under tumor sphere condition in the presence of STATTIC, an inhibitor of STAT3 signaling. The result revealed that STATTIC significantly reduced the number of tumor sphere formation by ADV-infected primary glioma cells, as well as the protein level of stemness-related transcription factors (Fig. 4e-g). These data suggested that ADV infection could promote the formation of GSCs from primary glioma cells by activation of TLR9-MYD88-STAT3 signaling.

### LncRNA NEAT1 is required for the formation of ADV-induced GSCs

**Fig. 5:**
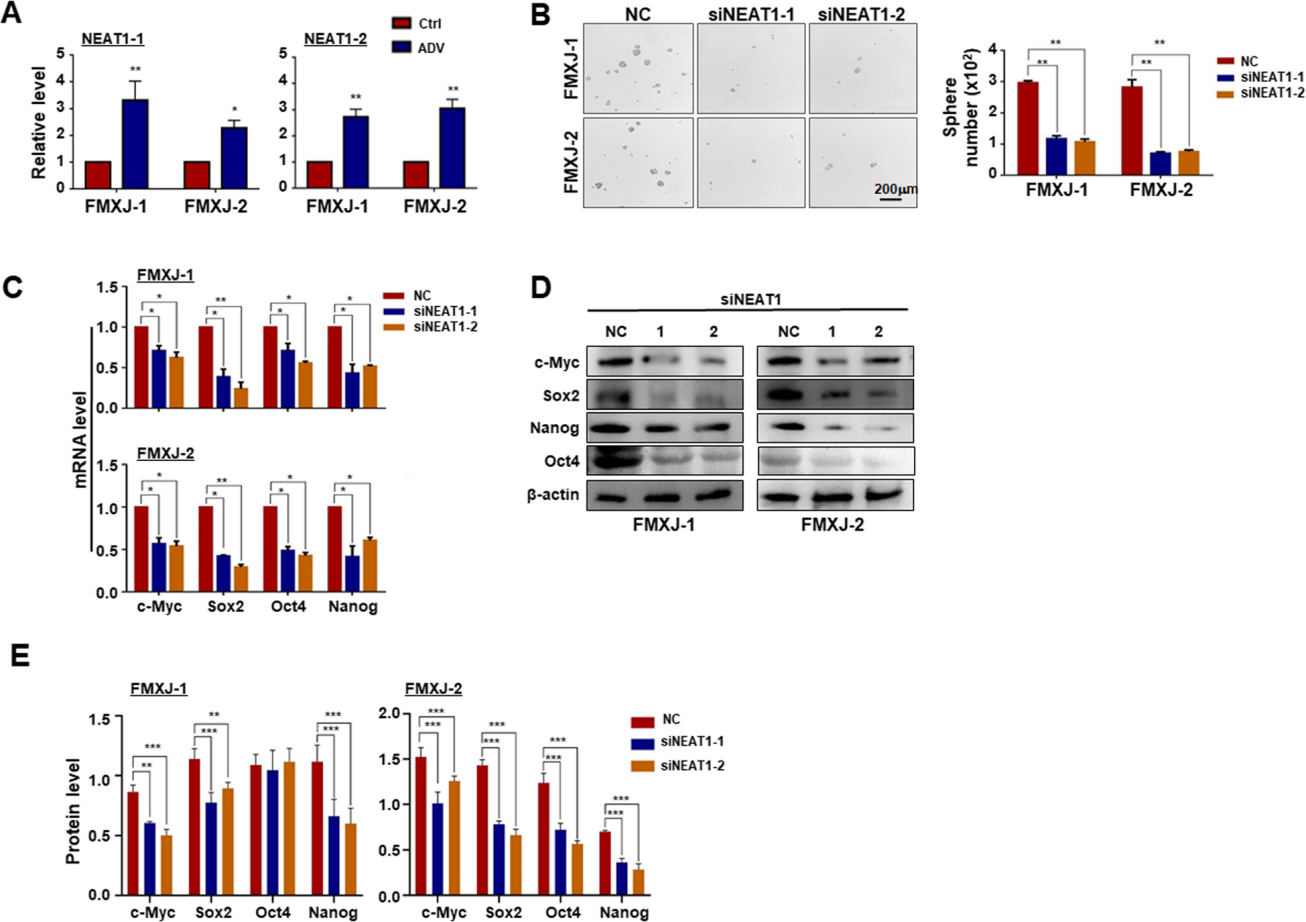
NEAT1 is upregulated and essential for ADV-induced GSCs formation by primary glioma cells. **a** Primary glioma cells were infected with ADV and cultured for 3 days. The expression of NEAT1 was determined by RT-qPCR, with β-actin as a reference control. **b** Primary glioma cells were infected with ADV and transfected with siRNAs to NEAT1, and then cultured under the neurosphere condition. The formation of tumorsphere was determined. **c-e** Primary glioma cells were infected with ADV and transfected with NEAT1 siRNAs, and then cultured for 3 days (NC, negative control. 1, siRNA-1. 2, siRNA-2). The expression of stemness-related transcription factors including c-MYC, SOX2, OCT4 and NANOG, was determined by RT-qPCR **c** and western blotting **d, e**, with β-actin as a reference control. Data are represented as mean ± SEM, *n* = 6. *, *P* < 0.05. **, *P* < 0.01. ***, *P* < 0.001

Recent studies have shown that ADV infection upregulates a panel of long noncoding RNA (lncRNA), which are downstream to TLRs and associated with stemness [45, 46]. We therefore determined the expression of lncRNA NEAT1, which is a core component of paraspeckles and is associated with cancer stem-like cells [31–34, 47], in primary glioma cells and ADV-induced tumor spheres by RT-qPCR. The result showed that the level of NEAT1 increased after ADV infection (Fig. 5a). To verify a potential role of NEAT1 in ADV-induced GSCs, siRNAs targeting NEAT1 was synthesized (supplementary Figure S4E). The result showed that knockdown of NEAT1 by siRNA reduced tumor sphere formation induced by ADV infection (Fig. 5b). The expression of stemness-related transcription factors was also downregulated by NEAT1 siRNA at both mRNA and protein levels as determined by RT-qPCR and western blotting (Fig. 5c-e). These data revealed that NEAT1 is likely a critical mediator of ADV-induced GSC formation.

### NEAT1 is associated with activation of TLR-STAT pathway in GBM

**Fig. 6:**
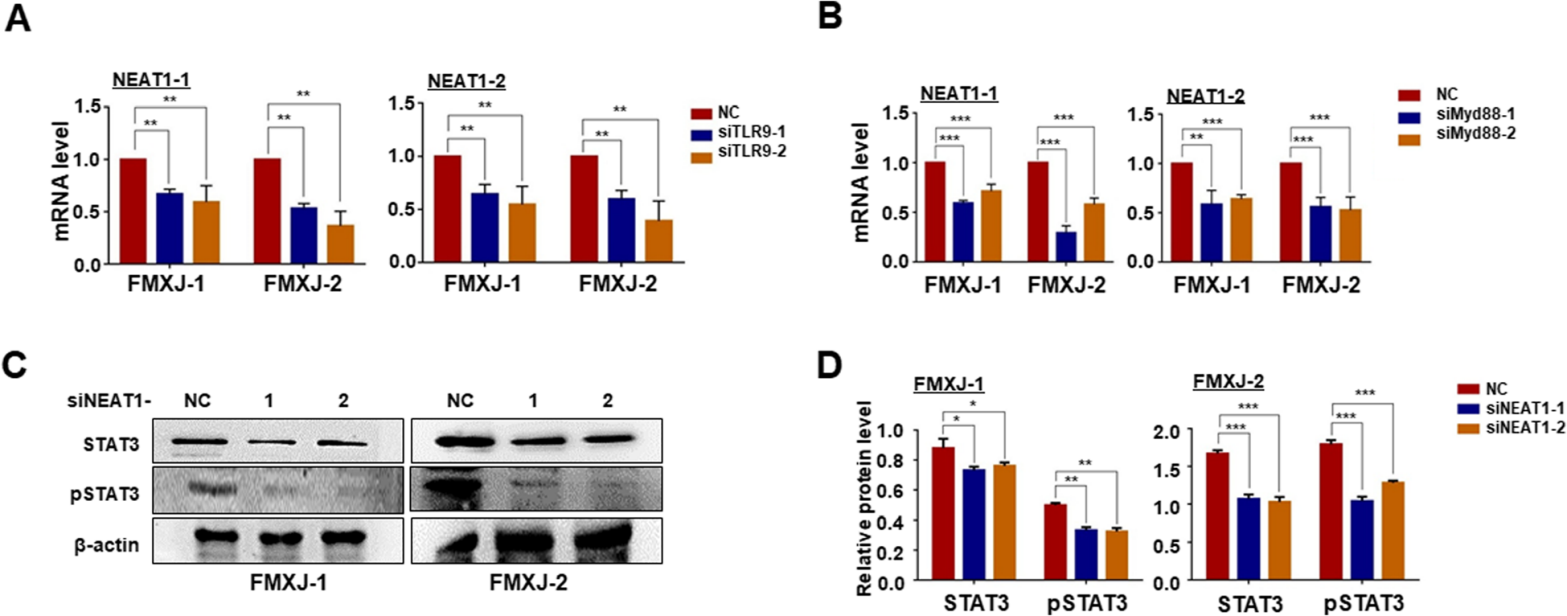
NEAT1 was downstream to TLR9-MYD88 and upstream to STAT3 in ADV-induced GSCs formation. **a** Primary glioma cells were infected with ADV and transfected with siRNAs to TLR9, and then the expression of NEAT1 was determined by RT-qPCR, with β-actin as a reference control. **b** Primary glioma cells were infected with ADV and transfected with siRNAs to MYD88, and then the expression of NEAT1 was determined by RT-qPCR, with β-actin as a reference control. **c, d** Primary glioma cells were infected with ADV and transfected with NEAT1 siRNAs, and then the level of STAT3 and p-STAT3 was determined by western blotting, with β-actin as a reference control (NC, negative control. 1, siRNA-1. 2, siRNA-2). Data are represented as mean ± SEM, *n* = 6. *, *P* < 0.05. **, *P* < 0.01. ***, *P* < 0.001

We then assessed the relationship between NEAT1 and the TLR9-STAT3 pathway using the siRNA tools. The results showed that downregulating TLR9 or MYD88 using siRNAs reduced the expression of NEAT1 after ADV infection (Fig. 6a, b). On the other hand, transfection of NEAT1 siRNA abrogated the increased levels of STAT3 and phosphorylated STAT3 induced by ADV infection (Fig. 6c, d, supplementary Figure S4F). These results, in combination with literatures, suggested that NEAT1 is downstream to TLR9-MYD88 and regulates STAT3 [46, 48–50].

### Activation of TLR9 potentially induces GSCs formation

**Fig. 7:**
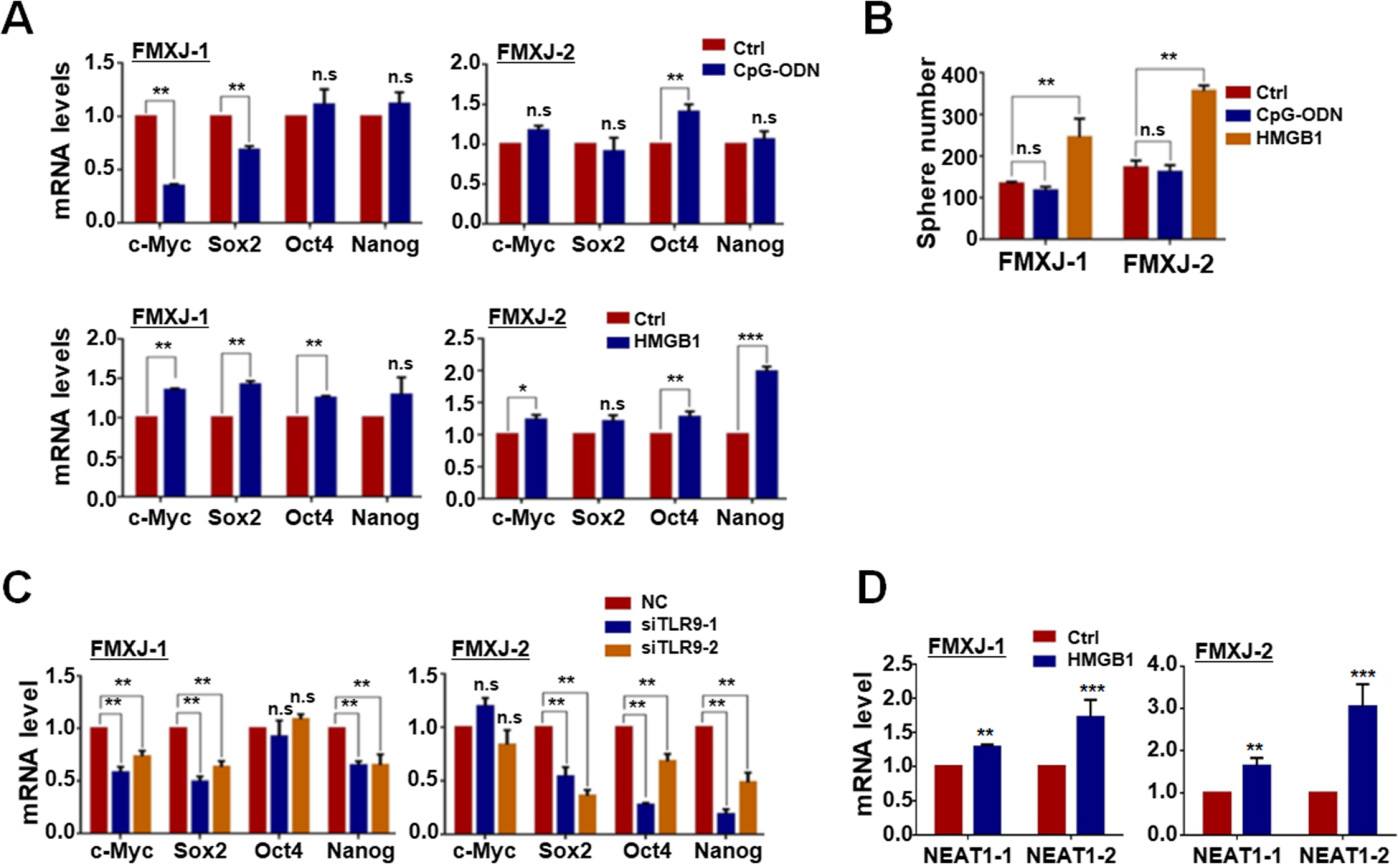
HMGB1, another DAMP, promoted GSCs formation by primary glioma cells. **a** Primary glioma cells were cultured in the presence of CpG-ODN or HMGB1. The expression of stemness-related transcription factors including c-MYC, SOX2, NANOG, and OCT4 was determined by RT-qPCR, with β-actin as a reference control. The following concentrations were used: HMGB1, 1 μg/ml; CpG-ODN, 2 μM. **b** Primary glioma cells were cultured under the neurosphere condition in the presence of HMGB1 (1 μg/ml) for 7 days and photographed. The number of tumor spheres was counted. **c** Primary glioma cells were cultured in the presence of HMGB1 (1 μg/ml) and transfected with siRNAs toTLR9. Cells were then cultured for 3 days. The expression of stemness-related transcription factors including c-MYC, SOX2, NANOG, and OCT4 was determined by RT-qPCR, with β-actin as a reference control. **d** Primary glioma cells were cultured in the presence of HMGB1 and the expression of NEAT1 was determined by RT-qPCR, with β-actin as a reference control. Data are represented as mean ± SEM, *n* = 6. *, *P* < 0.05. **, *P* < 0.01. ***, *P* < 0.001. n.s, not significant

TLR9 could be triggered by a panel of DAMPs such as CpG-containing oligodeoxyribonucleotides (CpG-ODN) and HMGB1, with the latter enriched in TME as an alarmin of tissue injury [24–26, 51]. We then tested the effects of these two TLR9 agonists on primary glioma cells. The result showed that while CpG-ODN did not significantly upregulate stemness-related transcription factors in primary glioma cells, HMGB1 did show this effect (Fig. 7a). Consistently, HMGB1 but not CpG-ODN increased the formation of tumor spheres by primary glioma cells (Fig. 7b). A correlation analysis of TCGA data also showed that HMGB1 level was positively correlated with the expression of SOX2 (supplementary Figure S4G). Similar as in ADV, HMGB1-induced upregulation of stemness-related transcription factors was attenuated by siRNAs targeting TLR9, confirming that TLR9 likely served as the receptor sensing HMGB1 (Fig. 7c). Moreover, the expression of NEAT1 was upregulated after HMGB1 treatment (Fig. 7d). Taken together, these results suggested that HMGB1 could promote the formation of GSCs by primary glioma cells via TLR9 and NEAT1.

## Discussion

Although the important roles of CSCs in cancer progression, recurrence and therapy-resistance have been well documented, the generation and behaviors of CSCs have largely not been defined. Classically, CSCs are considered as rare pathogenic stem cells, with clear properties of physiological stem cells, such as an intrinsic stem-maintenance machinery, slow amplification, asymmetric division to generate transient amplification cells that give rise to growing tumor [1]. However recent studies, by using advanced technologies such as single cell profiling, have also suggested another model that CSCs are plastic and transient cell states with stem cell properties [52–54]. This model appears to coincide better with at least some of the biological studies, which have revealed that various cues from TME could signal to and reprogram differentiated cancer cells into cells with certain extent of stemness. Our data reported in the current study added a novel environmental signal, ADV infection, that promotes the formation of GSCs from patient-derived primary glioma cells. The formation of GSCs from primary glioma cells is demonstrated by serial in vitro tumor sphere formation, expression of stemness-related transcription factors and CD133, as well as in vivo tumorigenesis assay. These observations might have clinical significance, because adenovirus occurrence has been demonstrated in pediatric tumor entities [55]. With a similar consideration, we have shown that HMGB1, a DAMP frequently generated in TME by tumor/TME cells under stress, also promoted GSCs formation from primary glioma cells. This might be involved in the generation of GSCs and likely other CSCs upon chemo- and radio-therapies, which always lead to tissue damage and release of DAMPs including HMGB1. However, we currently could not clarify ADV infection or HMGB1 treatment promotes dedifferentiation of differentiated glioma cells, or they promote the proliferation of rare GSCs in the stocks of patient-derived primary glioma cells. Moreover, given the heterogeneity of gliomas, it is worthwhile to note that ADV and other environmental cues may have variable effects on different patients and different disease stages, just like different response to ADV infection between FMXJ-1 and FMXJ-2.

The ADV used in the current study is a non-replicating one (pAdTrack-CMV) and expresses only GFP. ADV infection appears to promote GSCs by a single infection event without the expression of viral proteins or induction of cell death (data not shown). As a virus with a double-strand DNA genome, ADV components could be recognized by a series of host innate receptors including specific DNA sensor and TLRs [42–44]. Our data have shown that in glioma cells, ADV triggers TLR9 to induce GSCs formation. TLR9 is a nucleic acid recognition TLRs and is expressed both on endosomes and on cell surface after a complicated post-translational processing. When triggered by its ligands such as viral DNA, TLR9 signaling initiate immune response by producing cytokines and type I interferons through MYD88, which interacts with IL1R-associated kinase 4 (IRAK4) and activate NF-κB and the MAPKs pathways, and the type I IFN pathway [56]. These signaling pathways lead to and reinforce inflammatory responses in TME, and indirectly promote stemness of cancer cells [22]. However recently, TLR9 has been directly associated with CSCs including GSCs [27–29]. Gao et al. reported TLR9 signaling in TME initiates cancer recurrence after radiotherapy [27]. A short period of time later, two other groups demonstrated TLR9 is critical to the formation of CSCs in prostate cancer and GSCs [28, 29]. In both of these reports, the authors have shown that TLR9 participates in CSCs formation via STAT3, a multi-functional signal transduction molecule widely involved in stemness and cancer [30]. Our results are consistent with these findings and suggest that ADV infection could trigger TLR9-MYD88 signaling and lead to the formation of GSCs from primary glioma cells in a STAT3-dependent way. However, in our system, CpG-ODN appeared not able to trigger the stemness signaling in glioma cells, although it has been widely employed as an agonist of TLR9 [56]. More detailed dissecting of the signaling pathway downstream to TLR9 is required to clarify this inconsistency.

The lncRNA NEAT1 is an essential architectural component of paraspeckle nuclear bodies, and plays extensive tumorigenic roles in many types of human cancers [57, 58]. Dysregulated NEAT1 expression has been documented in a large panel of human cancers, and is associated with poor overall survival in these cancers [34]. Mechanistically, a lot of tumor- and TME-derived signals, such as hypoxia, EGFR-induced STAT3 and NF-κB activation, might be responsible for upregulated NEAT1 expression. The downstream effectors include a large array of miRNAs, leading to aberrant proliferation, migration/invasion, metastasis and chemo−/radio-resistance [34]. Expectedly, NEAT1 also plays a critical role in the generation and maintenance of CSCs [32, 33]. In GBM, NEAT1 is a critical effector of tumorigenesis and progression [13, 31]. Zhou et al. reported that Galectin-3 activates TLR4/NF-κB signaling to promote lung adenocarcinoma cell proliferation through activating lncRNA-NEAT1 expression [46]. In our study, the expression of NEAT1 was upregulated by ADV infection, and knocking down NEAT1 could suppress the ADV-induced GSCs formation. Mechanistically, upregulation of NEAT1 in GSCs under ADV infection could be attributed to TLR9 activation, and knockdown of NEAT1 was accompanied with an inhibition of STAT3 expression and phosphorylation. This is consistent with several reports showing that NEAT1 promotes cancer progression by enhancing STAT3 signaling [48–50]. Therefore, ADV infection likely triggers TLR9/MYD88 signaling, which upregulated NEAT1 and activated STAT3, and activated STAT3 further upregulates NEAT1 to form a positive-feedback loop, and promotes GSCs formation from primary glioma cells.

Adenovirus has been progressively modified to satisfy the needs of human gene therapy of cancer [59]. Moreover, the oncolytic virotherapy mostly employs adenovirus as the backbone to develop therapeutic vectors [60]. To achieve more specific tumor-targeting and better therapeutic effects, several modifications to the adenovirus genome have been made to enhance their oncolytic activity and ensure patient safety. For example, several groups have reported developing cancer-targeting adenovirus vectors by using libraries that display random peptides on a fiber knob [60]. On the other hand, a few disadvantages of adenovirus vectors have also been documented. Systemic administration of adenoviruses results in hepatic tropism independent of the primary receptors. Adenoviruses induce strong innate and acquired immunity in vivo [61]. Data reported in the current study raise another concern of using adenovirus vectors in cancer treatment, i.e., inducing the formation of CSCs. It would be required to elucidate the critical element in adenovirus to induce stemness of cancer cells. Then it could be achieved by genomic modification to overcome this potential problem in gene and/or oncolytic therapy.

## Conclusion

In summary, we have found that infection of patients-derived glioma cells with ADV increase the formation of tumor spheres. These tumor spheres express stem cells markers, hold the capacities of self-renewal and multi-lineage differentiation, and have stronger potential to form tumors after inoculated in immune-compromised mice. Mechanistically, these ADV-induced GSCs upregulated lncRNA NEAT1, which is downstream to TLR9 and plays important roles in cancer stem cells likely via strengthening STAT3. These discoveries suggest that ADV, as vector for gene therapy and oncolytic virus, could promote the formation of GSCs via TLR9/Neat1/Stat3 signaling.

## Supplementary information

**Additional file 1: Supplementary Table S1.** Primer sequences used for RT-qPCR analysis.

**Additional file 2: Supplementary Figure S1.** ADV promoted formation of tumor spheres from primary and lined glioma cells. Primary glioma cells FMXJ-2 and glioma cells lines A172 and T98G were infected with ADV and cultured for 7 days under tumor sphere condition. Cells were photographed on day 1 and day 7 of the culture.

**Additional file 3: Supplementary Figure S2.** In vivo tumorigenesis by GSCs derived from ADV-infected primary glioma cells. (A) Experimental design to evaluate in vivo tumorigenesis by GSCs derived from ADV-infected glioma cells. (B) Intracranial tumor formation by luciferase-labeled GSCs in nude mice as determined by bioluminescence using an IVIS Kinetic Imager. Different numbers of GSCs (500, 5000, 10,000 cells) were inoculated with 10,000 of primary glioma cells as a control (Ctrl). (C) Histology (H&E staining) of xenograft tumors from FMXJ-1 (5000 cells at initial inoculation). Pictures with different magnifications are shown. The arrow indicates an area of mitotic cells.

**Additional file 4: Supplementary Figure S3.** Identification of TLR9 as a mediator of ADV-induced GSCs. (A) Quantitative RT-PCR was performed to determine the expression of different DNA sensors in ADV-transfected primary glioma cells. (B, C) Primary glioma cells were transfected with siRNA to TLR9 or Myd88, and the expression of TLR9 and Myd88 was determined by western blotting and quantitatively compared. (D) Primary glioma cells were infected with ADV, and transfected with siRNAs to TLR9 or NC control. Tumor spheres were photographed after cultured for 7 days. (E) Primary glioma cells were infected with ADV, and transfected with siRNAs to TLR9 or NC control. The expression of Myd88 was determined by western blotting. (F) Level of p-STAT3 in relative to STAT3 in cells treated with siRNA to Myd88. Bars = mean ± SEM, *n* = 6. **, *P* < 0.01; ***, *P* < 0.001; n.s, not significant.

**Additional file 5: Supplementary Figure S4.** Analyses of signaling molecules leading to GSCs formation after ADV infection. (A) GSEA analysis of the enrichment of molecules involved in JAK-STAT signaling and TLR signaling using glioma data sets from TCGA and CGGA. (B) Kaplan-Meier plots of GBM patients data in TCGA based on MYD88 and STAT3 expression. Patients were separated based on median expression values. (C, D) Western blotting to determine the levels of Myd88, Stat3 and phosphorylated Stat3 in ADV-infected and control primary glioma cells. Data were quantitatively compared in (D). (E) RT-qPCR to determine the suppressing efficiency of siRNAs targeting NEAT1. (F) Level of p-STAT3 in relative to STAT3 in cells treated with siRNA to NEAT1. (G) Correlation between HMGB1 level and SOX2 expression was analyzed using the TCGA database. Bars = mean ± SEM, n = 6. *, *P* < 0.05; **, *P* < 0.01; ***, *P* < 0.001; n.s, not significant.

## Data Availability

Not applicable.

## Abbreviations

GSCs: Glioma stem cells
TME: Tumor microenvironment
ADV: Adenovirus
DAMP: Damage associated molecular pattern
HMGB1: High mobility group box 1
TLR9: Toll-like receptor 9
MYD88: Myeloid differentiation primary response gene 88
STAT3: Signal transducer and activator of transcription 3
NEAT1: Nuclear enriched abundant transcript 1
GBM: Glioblastoma multiforme
FBS: Fetal bovine serum
H&E: Hematoxylin and eosin
PFA: Paraformaldehyde
BSA: Bovine serum albumin
MAP2: Mitogen associated protein 2
GFAP: Gial fibrilling acidic protein
SDS-PAGE: Sodium dodecyl sulfate-polyacrylamide gel electrophoresis
PVDF: Electro-transferred onto polyvinyl difluoride
GSEA: Gene set enrichment analysis
NES: Normalized enrichment scores
SEM: Mean ± standard error of mean
ANOVA: Analysis of Variance
CpG-ODN: CpG-containing oligodeoxyribonucleotides
IRAK4: IL1R-associated kinase 4
