# Supplementary material for: Adenovirus infection promotes the formation of glioma stem cells from glioblastoma cells through the TLR9/NEAT1/STAT3 pathway
Source: Cell Commun Signal. 2020 Aug 26;18:135. doi: 10.1186/s12964-020-00598-7 (PMC7448505; doi:10.1186/s12964-020-00598-7)
Supplement: Supplementary file 2 — Additional file 1: Supplementary Table S1. Primer sequences used for RT-qPCR analysis. [file 12964_2020_598_MOESM2_ESM.docx]

**Supplementary materials**

**Supplementary Table S1. Primer sequences used for qRT-PCR analysis.**

| Gene | Forward | Reverse |
| --- | --- | --- |
| β-actin | 5’- AGAAAATCTGGCACCACACC-3’ | 5’-AGAGGCGTACAGGGATAGCA-3’ |
| c-MYC | 5’-AATGAAAAGGCCCCCAAGGTAG-3’ | 5’-GTCGTTTCCGCAACAAGTCCT-3’ |
| SOX2 | 5'-CACACTGCCCCTCTCAC-3' | 5'-TCCATGCTGTTTCTTACTCTCC-3' |
| OCT4 | 5’-TCTCCCATGCATTCAAACTGAG-3’ | 5’-CCTTTGTGTTCCCAATTCCTTC-3’ |
| NANOG | 5’-GAAATACCTCAGCCTCCAGC-3’ | 5’-GCGTCACACCATTGCTATTC-3’ |
| DANCR | 5’-GCGCCACTATGTAGCGGGTT-3’ | 5’-TCAATGGCTTGTGCCTGTAGTT-3’ |
| FENDRR | 5’-AGACAAAAACTCACTGCCCA-3’ | 5’-TGATGTTCTCCTTCTTGCCTC-3’ |
| XIST | 5’-ACGCTGCATGTGTCCTTAG-3’ | 5’-GAGCCTCTTATAGCTGTTTG-3’ |
| FTX | 5’-CAAAGCTGGTCCTGTGCCTG-3’ | 5’-ATTGAGTGTGGCATCACCTCC-3’ |
| NEAT1-1 | 5’-CCAGTTTTCCGAGAACCAAA-3’ | 5’-ATGCTGATCTGCTGCGTATG-3’ |
| NEAT1-2 | 5’-CTAGAGGCTCGCATTGTGTG-3’ | 5’-GCCCACACGAAACCTTACAT-3’ |
| TLR1 | 5’-CAAATGGAACAGACAAGCAGG-3’ | 5’-GCCTGGTACCCCTATTAGTG-3’ |
| TLR2 | 5’-AGACCTATAGTGACTCCCAGG-3’ | 5’-ACCCACACCATCCACAAAG-3’ |
| TLR3 | 5’-TCAACTTTCTGATAAAACCTTTGCC-3’ | 5’-AGATGACAAGCCATTATGAGACA-3’ |
| TLR4 | 5’-TGCGTGAGACCAGAAAGC-3’ | 5’-TTAAAGCTCAGGTCCAGGTTC-3’ |
| TLR5 | 5’-TCCAGGGTTCAAGCGATTC-3’ | 5’-CGTTGTCAGTAGCATCAGGAG-3’ |
| TLR6 | 5’-TGGACTCATATCAAGATGCTCTG-3’ | 5’-GTCGGAGAACTGGATTCTGG-3’ |
| TLR7 | 5’-GAAAGTTGATGCTATTGGGCC-3’ | 5’-GAATTTGTCTCTTCAGTGTCCAC-3’ |
| TLR8 | 5’-CTGCATAGAGGGTACCATTCTG-3’ | 5’-CGCATAACTCACAGGAACCAG-3’ |
| TLR9 | 5’-CTATAACCGGAACTTCTGCCAG-3’ | 5’-CTGCTCTGTGTCAGGTGTG-3’ |
