# Supplementary figures and images for: Adenovirus infection promotes the formation of glioma stem cells from glioblastoma cells through the TLR9/NEAT1/STAT3 pathway
Source: Cell Commun Signal. 2020 Aug 26;18:135. doi: 10.1186/s12964-020-00598-7 (PMC7448505; doi:10.1186/s12964-020-00598-7)

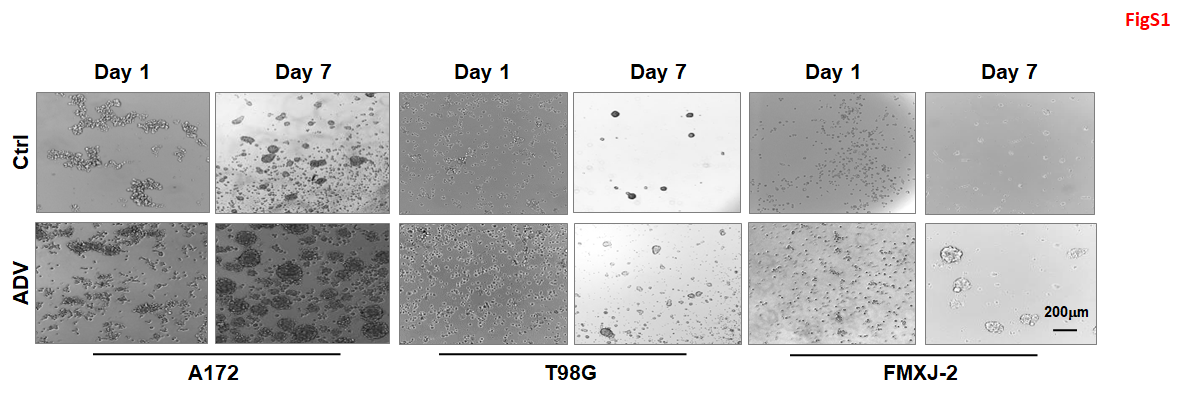

Supplement: Supplementary file 3 — Additional file 2: Supplementary Figure S1. ADV promoted formation of tumor spheres from primary and lined glioma cells. Primary glioma cells FMXJ-2 and glioma cells lines A172 and T98G were infected with ADV and cultured for 7 days under tumor sphere condition. Cells were photographed on day 1 and day 7 of the culture. [file 12964_2020_598_MOESM3_ESM.tif]

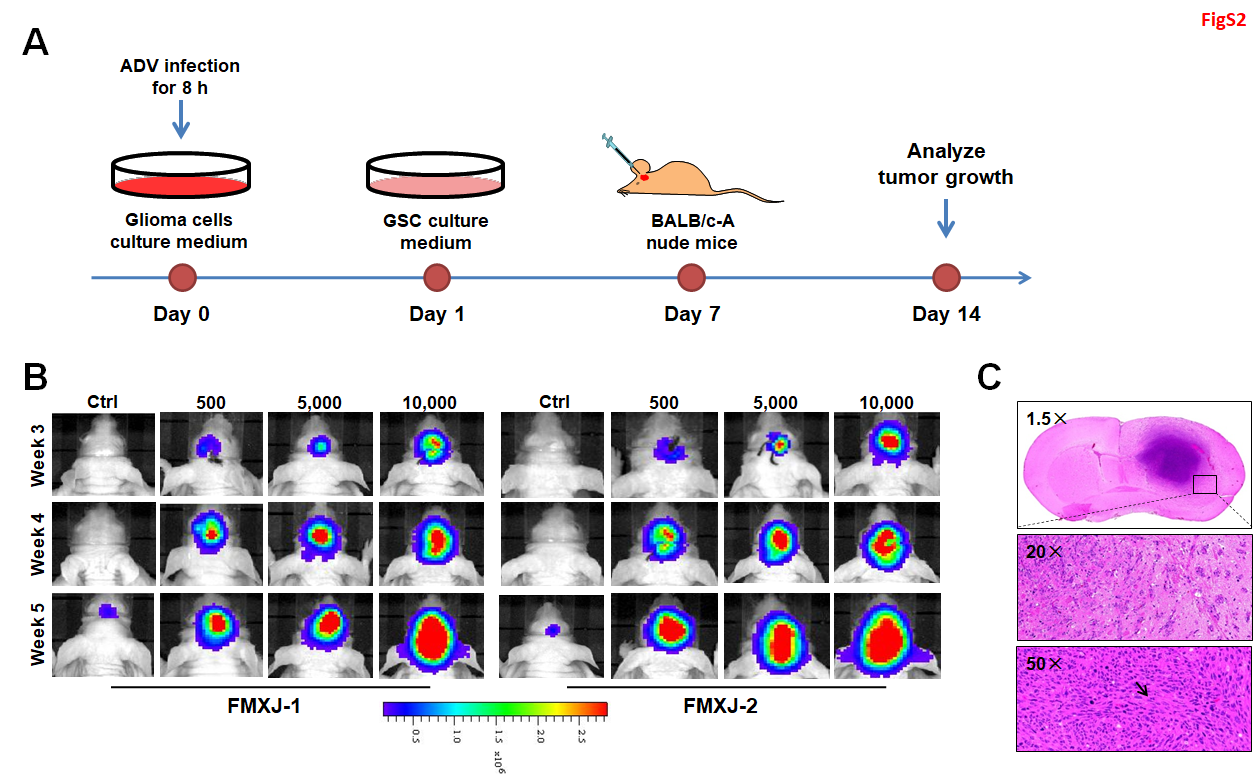

Supplement: Supplementary file 4 — Additional file 3: Supplementary Figure S2. In vivo tumorigenesis by GSCs derived from ADV-infected primary glioma cells. (A) Experimental design to evaluate in vivo tumorigenesis by GSCs derived from ADV-infected glioma cells. (B) Intracranial tumor formation by luciferase-labeled GSCs in nude mice as determined by bioluminescence using an IVIS Kinetic Imager. Different numbers of GSCs (500, 5000, 10,000 cells) were inoculated with 10,000 of primary glioma cells as a control (Ctrl). (C) Histology (H&E staining) of xenograft tumors from FMXJ-1 (5000 cells at initial inoculation). Pictures with different magnifications are shown. The arrow indicates an area of mitotic cells. [file 12964_2020_598_MOESM4_ESM.tif]

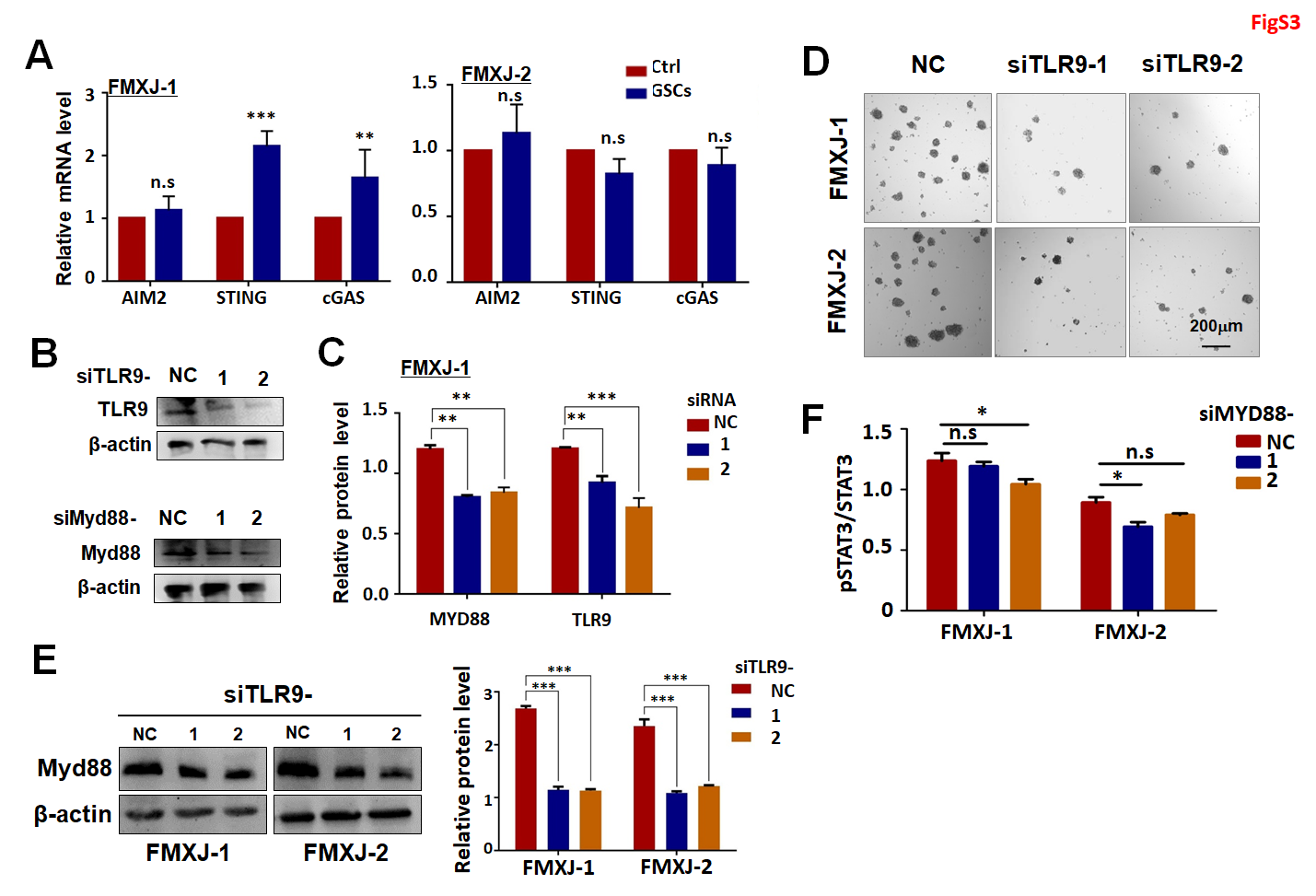

Supplement: Supplementary file 5 — Additional file 4: Supplementary Figure S3. Identification of TLR9 as a mediator of ADV-induced GSCs. (A) Quantitative RT-PCR was performed to determine the expression of different DNA sensors in ADV-transfected primary glioma cells. (B, C) Primary glioma cells were transfected with siRNA to TLR9 or Myd88, and the expression of TLR9 and Myd88 was determined by western blotting and quantitatively compared. (D) Primary glioma cells were infected with ADV, and transfected with siRNAs to TLR9 or NC control. Tumor spheres were photographed after cultured for 7 days. (E) Primary glioma cells were infected with ADV, and transfected with siRNAs to TLR9 or NC control. The expression of Myd88 was determined by western blotting. (F) Level of p-STAT3 in relative to STAT3 in cells treated with siRNA to Myd88. Bars = mean ± SEM, n = 6. **, P < 0.01; ***, P < 0.001; n.s, not significant. [file 12964_2020_598_MOESM5_ESM.tif]

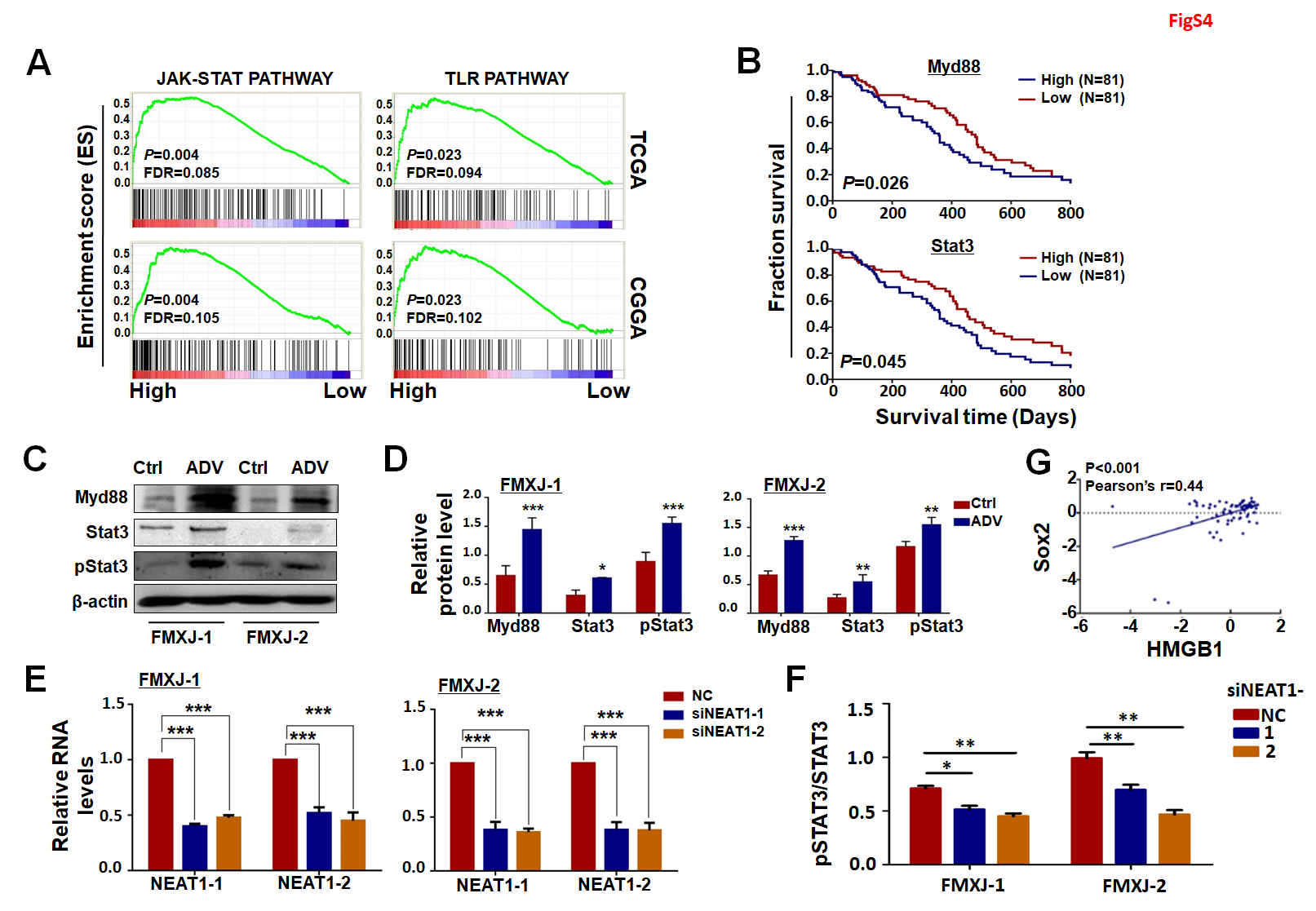

Supplement: Supplementary file 6 — Additional file 5: Supplementary Figure S4. Analyses of signaling molecules leading to GSCs formation after ADV infection. (A) GSEA analysis of the enrichment of molecules involved in JAK-STAT signaling and TLR signaling using glioma data sets from TCGA and CGGA. (B) Kaplan-Meier plots of GBM patients data in TCGA based on MYD88 and STAT3 expression. Patients were separated based on median expression values. (C, D) Western blotting to determine the levels of Myd88, Stat3 and phosphorylated Stat3 in ADV-infected and control primary glioma cells. Data were quantitatively compared in (D). (E) RT-qPCR to determine the suppressing efficiency of siRNAs targeting NEAT1. (F) Level of p-STAT3 in relative to STAT3 in cells treated with siRNA to NEAT1. (G) Correlation between HMGB1 level and SOX2 expression was analyzed using the TCGA database. Bars = mean ± SEM, n = 6. *, P < 0.05; **, P < 0.01; ***, P < 0.001; n.s, not significant. [file 12964_2020_598_MOESM6_ESM.tif]
